# Management of emotionally unstable personality disorder in an urban Irish setting

**DOI:** 10.1192/bjo.2021.854

**Published:** 2021-06-18

**Authors:** Calvin Har Siu Yee

**Affiliations:** St. Vincent's Hospital

## Abstract

**Aims:**

To determine the prevalence of emotionally unstable personality disorder (EUPD) attending a community mental health team (CMHT) in a major Irish city

To describe the current psychiatric care afforded to this cohort of service user

**Method:**

Clinical chart review of all 328 patients attending a CMHT outpatient in an urban setting was carried out. Patients diagnosed with EUPD or displayed features of EUPD were identified. Data on the various interventions offered to this cohort of service users were collected and compared against current guidelines.

**Result:**

Out of the 328 patients actively attending the service, almost 17% (n = 55) were diagnosed with EUPD and further 6% (n = 19) were found to display features of EUPD such as emotional dysregulation, self-harming behaviour and cognitive distortions. Comorbid psychiatric disorder such as mood or anxiety spectrum disorder was diagnosed in 23% (n = 17) of this cohort. Meanwhile, 8% (n = 6) was diagnosed with addiction disorders and 5% (n = 4) was diagnosed with a comorbid personality disorder. A significant proportion of 77% (n = 57) were prescribed psychotropic medication with 51% (n = 29) being on more than one psychotropic medication. Antidepressants, antipsychotics and hypnotics were the three most common medications prescribed at the rate of 89% (n = 51), 30% (n = 17) and 28% (n = 16) respectively. A majority of 66% (n = 49) were offered intervention from a multi-disciplinary team (MDT) member with 47% (n = 23) being offered more than one type of intervention. Referrals to community mental health nurses and psychology service were the two most common interventions offered with a referral rate of 59% (n = 29) and 55% (n = 27) respectively. 28% (n = 21) of service users with EUPD or EUPD traits has had at least one hospital admission while attending the CMHT and 46% (n = 34) have been admitted to the day hospital at least once.

**Conclusion:**

The prevalence of EUPD in our outpatient sample corresponds with findings in previous studies. Standard psychiatric care is the most common option available to the majority of general adult patients with EUPD in Ireland due to the lack of any national treatment programme and scarce availability of specialised therapeutic approaches such as dialectical behavioural therapy within community mental health teams. Our CMHT will attempt to integrate mentalization-based treatment into our outpatient management of EUPD patients taking into account current clinical guidelines for management of EUPD and resources needed for training and delivering the intervention.

